# Analysis on GENIE reveals novel recurrent variants that affect molecular diagnosis of sizable number of cancer patients

**DOI:** 10.1186/s12885-019-5313-1

**Published:** 2019-02-01

**Authors:** Takahiko Koyama, Kahn Rhrissorrakrai, Laxmi Parida

**Affiliations:** IBM TJ Watson Research Center, Yorktown Heights, NY 10598 USA

**Keywords:** Precision medicine, GENIE, Recurrent variants, Variant of unknown significance, Variant disparity

## Abstract

**Background:**

Significant numbers of variants detected in cancer patients are often left labeled only as variants of unknown significance (VUS). In order to expand precision medicine to a wider population, we need to extend our knowledge of pathogenicity and drug response in the context of VUS’s.

**Methods:**

In this study, we analyzed variants from AACR Project GENIE Consortium APG (Cancer Discov 7:818-831, 2017) and compared them to the COSMIC database Forbes et al. (Nucleic Acids Res 43:D805-811, 2015) to identify recurrent variants that would merit further study. We filtered out known hotspot variants, inactivating variants in tumor suppressors, and likely benign variants by comparing with COSMIC and ExAC Lee et al. (Science 337:967-971, 2012).

**Results:**

We have identified 45,933 novel variants with unknown significance unique to GENIE. In our analysis, we found on average six variants per patient where two could be considered as pathogenic or likely pathogenic and the majority are VUS’s. More importantly, we have discovered 730 recurrent variants that appear more than 3 times in GENIE but less than 3 in COSMIC. If we combine the recurrences of GENIE and COSMIC for all variants, 2586 are newly identified as occurring more than 3 times than when using COSMIC alone.

**Conclusions:**

Although it would be inappropriate to blindly accept these recurrent variants as pathogenic, they may warrant higher priority than other observed VUS’s. These newly identified recurrent variants might affect the molecular profiles of approximately 1 in 6 patients. Further analysis and characterization of these variants in both research and clinical contexts will improve patient treatments and the development of new therapeutics.

**Electronic supplementary material:**

The online version of this article (10.1186/s12885-019-5313-1) contains supplementary material, which is available to authorized users.

## Background

In cancer genomic analysis, it is commonplace to find rare variants whose pathogenicity and contributions to various aspects of tumorigenesis are not easily evaluated. In those circumstances, such variants are labeled variants of unknown significance (VUS) and focus is shifted to pathogenic or likely pathogenic mutations. Since the bulk of variants are VUS’s, there are many efforts to characterize them by using functional cell-based assays, somatic mutation signatures [1], gene expression [2], and structure based approaches [3]. Although functional cell-based assay approaches are powerful, they are time consuming and can still fall short of capturing certain aspects of pathogenicity, particularly those of a multicellular nature, such as escape from the immune system. Somatic signature and gene expression analysis requires whole genome or exome sequencing and gene expression data that are not attainable from panel sequence assays, which are the most commonly performed assay in the clinic today. Instead by studying the characteristics of variants observed over many panel sequenced samples, it may be possible to better understand the relevancy of a particular alteration.

An oft used metric for prioritizing VUS’s is the recurrence rate. Although high recurrence is insufficient to indicate pathogenicity, it can assist doctors in hypothesizing as to the etiological cause of the tumor and highlight specific VUS’s. Databases like COSMIC [4] and cBioPortal [5] are cataloging variants observed in a wide variety of studies. By offering a comprehensive set of observed alterations, researchers and clinicians can better prioritize variants in their own samples for further study or action, particularly when the exact biological function is unclear. By studying the frequencies, distributions, and types of variants seen across many cancers and associated clinical information, it may be possible to better classify a novel variant and advance precision medicine through the development of more accurate diagnostic, prognostic, and therapeutic markers and signatures.

AACR’s GENIE project [6] is a multi-year study to advance precision oncology. By working with cancer centers around the world, GENIE has collected genomic and clinical data from tens of thousands of cancer patients. Such a project is vital to improving the identification of actionable variants, particularly in light of the high variability in detecting actionable variants found across smaller studies. A recent precision medicine study shows that only 10 % of patients are eligible for FDA-labeled targeted treatment [7]. However, approximately half of patients had actionable variants in the MOSCATO 01 trial [8]. By performing a broad variant analysis on this new resource, we hope to characterize a set of novel and potentially clinically relevant VUS’s to enable precision medicine to better address a wider patient population. Such recurrent variants would serve as new lines of research inquiry and better enable clinicians to assess and act upon the genomic profile of their own patients.

## Methods

**Fig. 1:**
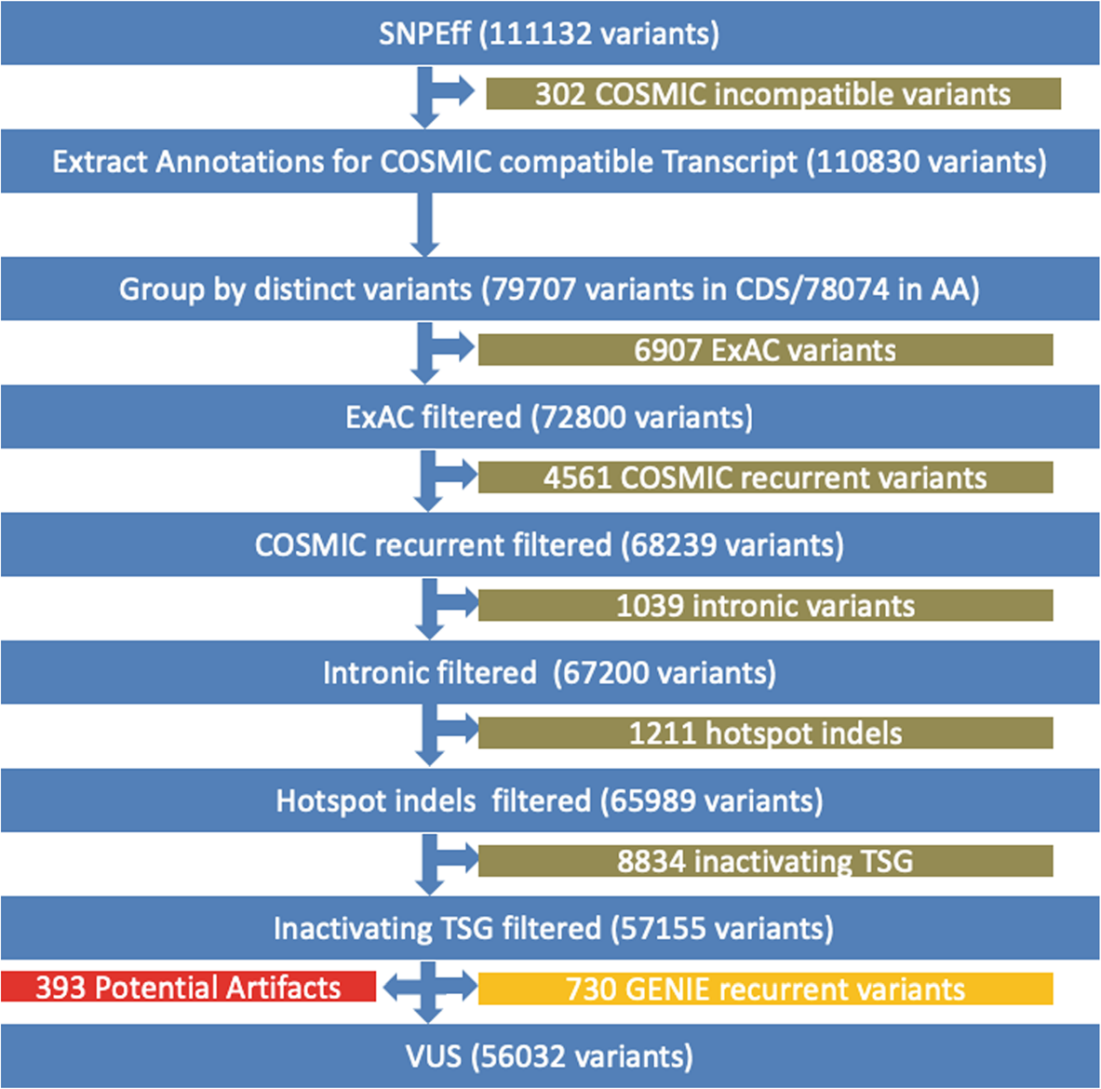
Process flow diagram of filters to remove variants

GENIE ver. 1.0, publicly released on January 5th, 2017, was used for this study. Samples in 524 tumor types from 32 tissues including both liquid and solid malignancies were sequenced at 8 participating centers using 12 cancer panels [9]. Dana Farber Cancer Institute, Memorial Sloan Kettering Cancer Center, and Vanderbilt-Ingram Cancer Center used hybridization capture whereas the remaining five centers used a PCR method. Not all panels included full genes with promoters and introns, and some only cover hotspots. Most tumor samples are not accompanied with matching normal samples except those from Memorial Sloan Kettering Cancer Center and Vanderbilt-Ingram Cancer Center; thus, it is important to remove potential germline variants. GENIE provides neither copy number alteration nor structural variants; therefore, this study focuses on recurrent SNV and small indels. The workflow of various filters to classify variants and to extract GENIE recurrent variants is illustrated (Fig. 1).

SNPEff [10] ver 4.3 using GRCh37.75 database was used to annotate variants. SNPEff annotations were extracted for COSMIC compatible transcripts. Although many COSMIC transcripts were consistent with Ensembl transcript IDs, some were provided as a RefSeq transcript ID, had been deprecated or belonged to non-human organisms. These inconsistencies were manually corrected; however, there remained several transcripts that could not be matched with any COSMIC transcripts.

ExAC release 0.3.1 [11] was downloaded and adjusted allele counts (AC_adj) and adjusted total counts (AN_adj) were extracted for each variant. Although the GENIE dataset already had some variant filtering using ExAC, there remained alterations that appeared with higher than expected frequency in the ExAC database. After application of Hypothesis Testing for the Difference in Population Proportions with 5% (Z > = 1.645) significance level, 6907 variants are removed.

Besides transcript compatibility issues, there were other challenges in comparing variants between GENIE and COSMIC. There were slight differences in the notation of variants between COSMIC and SNPEff outputs. For instance, SNPEff duplication annotations like p.L23dup is not used in COSMIC and instead COSMIC uses *ins* rather than *dup*. Also, SNPEff promoter variants such as c.-124C > T are expressed as c.1-124C > T in COSMIC. A tandem double variant in COSMIC may be expressed as c.1798_1799GT > AA whereas SNPEff outputs it as c.1798_1799delGTinsAA. As for amino acid change notations, the SNPEff deletion p.G469del might be written as p.G469delG in COSMIC. Finally, COSMIC has many instances of “c.?”, representing an unknown coding sequence change. After resolving these issues, we successfully removed 4561 COSMIC recurrent variants with counts ≥3.

Further filtering steps included removing intronic variants, short indels in hotspots, and inactivating variants in tumor suppressor genes. Intronic variants located 2 bp outside of the exon boundary were excluded but critical splicing acceptor and donor variants were kept. Upstream and downstream variants beyond 1000 bp from start and stop codon were discarded. These steps resulted in 1039 variants being removed. Short indels in hotspots were filtered out. If we observed more than 10 overlapping indels in a region regardless of being in-frame or not, we deemed the region a hotspot. Well known regions in cancer genomics include PIK3R1 (p85alpha iSH2 domain) [12], FLT3 ITD near R595 (Y591 and Y597) in exon 14 [13], and EGFR exon 19 [14] and exon 20 [15]. 1211 hotspot indels were removed as a result.

Inactivating variants such as stop gained, start loss, frameshift, splicing acceptor, splicing donor, and stop loss were considered as likely loss of function, and when found in tumor suppressor genes, they were removed under the assumption they were likely pathogenic. We have manually annotated tumor suppressors for the 536 GENIE mutated genes. These include some unequivocal tumor suppressors accepted by many, such as TP53, RB1, PTEN, NF1, APC, and CDKN2A. Though less established, many other genes such as B2M [16], CBFB [17], CUL3 [18], FUBP1 [19], GATA3 [20], GPS2 [21],HLA-A [22], MAP3K1 [23], MGA [24], NCOR1 [25], RASA1 [26], RBM10 [27], RNF43 [28], and RYBP [29] were included based upon current evidence in the literature. The full list of tumor suppressor genes defined in this study and corresponding evidences to support their designations is provided in the supplementary material (Additional file 1: Table S1). Using this set of tumor suppressors, 8834 variants were removed by this filter.

There remains the possibility that a number of sequencing related artifacts may be present in the recurrent list. To minimize such artifacts, we removed variants found only from a single sequencing center and not listed in COSMIC. With these criteria and a frequency threshold of at least three samples, 730 recurrent variants unique to GENIE were discovered.

## Results

**Fig. 2:**
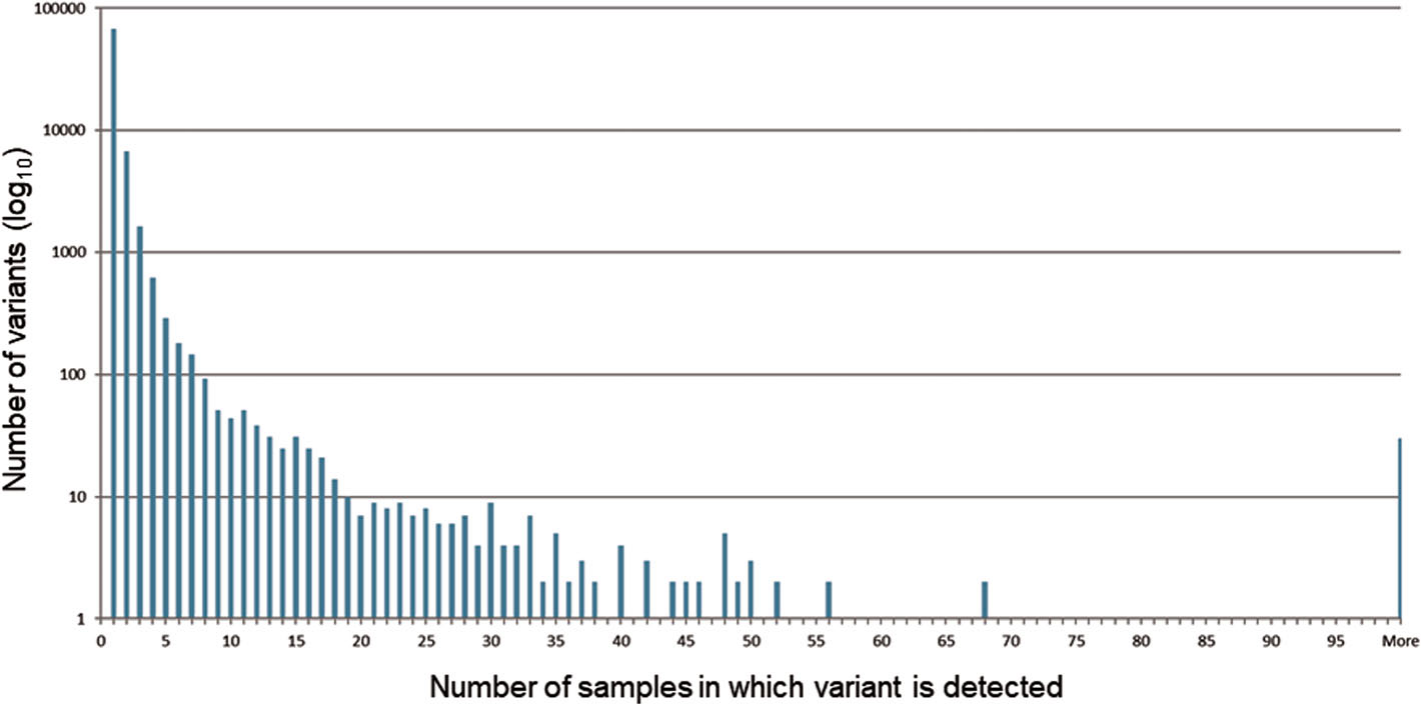
Variant recurrence in GENIE samples. Histogram indicates the number of variants (y-axis, log-scale) that occur at a given frequency (x-axis). As the frequency of recurrence increases, the number of variants decreases. However, a sizable number of variants observed in over 100 samples, which are listed in Table 1

The GENIE project contains data from 18,966 patients generated from a variety of sequence panels. A total of 111,132 variants were observed across these samples with a mean of six variants per sample. The processing of these variants is described in the methods. In brief, variants that do not lie within COSMIC gene transcripts were removed, leaving 110,830 variants. Among those, there are 79,707 coding sequence (CDS) changes and 78,074 variants leading to an amino acid change. 67,793 variants appeared only once in GENIE and 30 variants are observed over 100 times (Fig. 2).

These highly recurrent variants are mostly found in well-established cancer genes like KRAS, TP53, and PIK3CA. KRAS G12D was the most frequently observed (711 samples) followed by BRAF V600E (615 samples) (Table 1). There are hotspot variants found for individual cancers. In NSCLC, expected recurrent variants in KRAS, TP53, and PIK3CA are observed alongside hotspot variants EGFR L858R and exon 19 deletion E746_A750del. IDH codon 132 variants are seen in various cancers [30], and AKT1 E17K is commonly observed in breast cancer [31]. FGFR3 S249C often appears in bladder cancer [32]. All the highly recurrent variants are well known to the cancer community and are part of the hall of fame list.

**Table 1:** Hall of fame variants that appear in over 100 samples

Gene	AA change	CDS change	Counts
KRAS	p.Gly12Asp	c.35G > A	711
BRAF	p.Val600Glu	c.1799 T > A	615
KRAS	p.Gly12Val	c.35G > T	607
PIK3CA	p.Glu545Lys	c.1633G > A	524
PIK3CA	p.His1047Arg	c.3140A > G	500
KRAS	p.Gly12Cys	c.34G > T	449
TP53	p.Arg175His	c.524G > A	370
PIK3CA	p.Glu542Lys	c.1624G > A	332
IDH1	p.Arg132His	c.395G > A	323
KRAS	p.Gly13Asp	c.38G > A	260
TP53	p.Arg273His	c.818G > A	243
TP53	p.Arg273Cys	c.817C > T	242
TP53	p.Arg248Gln	c.743G > A	223
TP53	p.Arg248Trp	c.742C > T	211
TP53	p.Arg282Trp	c.844C > T	182
TP53	p.Arg213*	c.637C > T	179
NRAS	p.Gln61Arg	c.182A > G	173
EGFR	p.Leu858Arg	c.2573 T > G	160
EGFR	p.Glu746_Ala750del	c.2236_2250delGAATTAAGAGAAGCA	159
AKT1	p.Glu17Lys	c.49G > A	155
KRAS	p.Gly12Ala	c.35G > C	152
TP53	p.Arg342*	c.1024C > T	135
NRAS	p.Gln61Lys	c.181C > A	123
TP53	p.Arg196*	c.586C > T	110
IDH1	p.Arg132Cys	c.394C > T	109
KRAS	p.Gln61His	c.183A > T	108
TP53	p.Tyr220Cys	c.659A > G	108
APC	p.Arg1450*	c.4348C > T	102
APC	p.Arg876*	c.2626C > T	100
FGFR3	p.Ser249Cys	c.746C > G	100

Among the most frequently mutated genes, TP53 ranks highest with 8083 variants followed by KRAS with 2811 variants (Table 2). This set of highly mutated genes also contains many epigenetic regulators, such as KMT2D, ARID1A, KMT2A, ARID1B, ARID2, SMARCA4, TET2, ATRX, CREBBP, and EP300. For example, KMT2D, also known as MLL2, is a lysine methyl transferase that activates genes by methylating histone H3 at lysine 4 residue [33]. ARID1A is a SWI/SNF complex component that alters the expression of diverse genes through chromatin remodeling [34].

**Table 2:** Top mutated genes in GENIE

Gene	Variants observed
TP53	8083
KRAS	2811
PIK3CA	2693
APC	2674
KMT2D	1980
ARID1A	1494
PTEN	1313
EGFR	1212
ATM	1202
NF1	1122
BRAF	1116
BRCA2	949
NOTCH1	924
RB1	917
ATRX	887
SETD2	845
CREBBP	840
CDKN2A	788
ERBB4	740
KMT2A	739
SMAD4	733
ARID1B	719
SMARCA4	696
ROS1	688
FBXW7	687
EP300	669
ARID2	649
PTPRD	647
TET2	642
DNAPK	639
Others	70,732

**Fig. 3:**
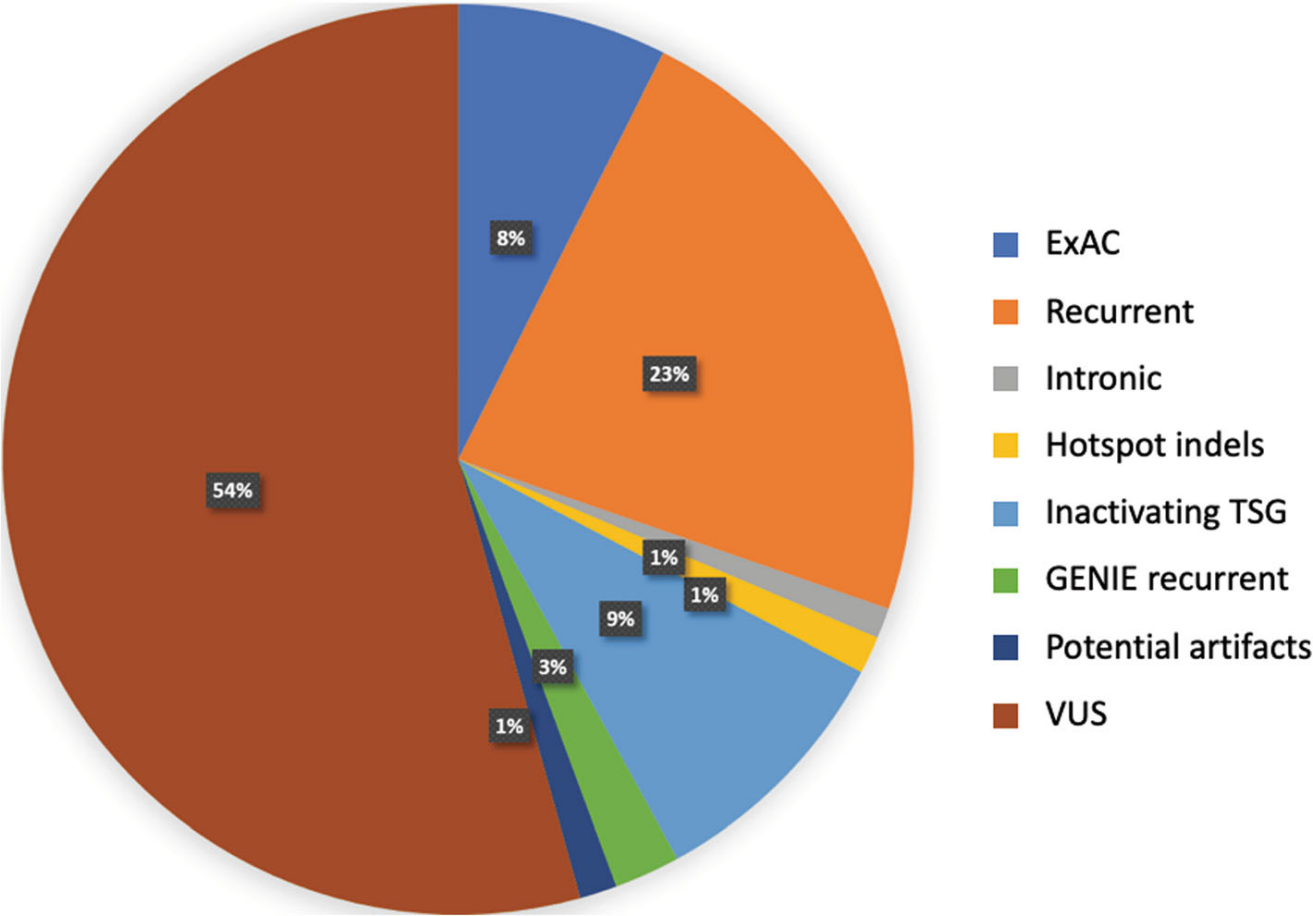
Variants classified according to filters. The percent of variants classified by each of the following filters: *ExAC* – variants with similar or higher frequencies in ExAC; *Recurrent* – variants detected in ≥3 samples in COSMIC; *Intronic* – variants found in introns excluding splice junctions; *Inactivating variant in TSG* – likely inactivating factors that occur in tumor suppressor gene; *GENIE recurrent* – variants detected in ≥3 samples in GENIE and < 3 samples in in COSMIC; *Potential artifacts* – variants occurring only from a single sequencing center; and *VUS* – all remaining variants are considered variants of unknown significance. Newly retrieved recurrent variants revealed in this study accounts for 3% (*GENIE recurrent*)

To further focus on coding VUS’s, we removed intronic variants, hotspot indels, inactivating variants in tumor suppressor genes, and variants according to their population frequencies. 6907 variants were filtered out by comparing variant frequencies between the ExAC database and GENIE (Table 3, Fig. 3) to remove variants observed in the general population at similar or higher rates than in GENIE. Following these filtering steps, 56,032 variants remained as VUS’s. Of the average six variants observed per patient, we found that approximately 1/3 are potentially significant as they are frequently mutated in cancer or are likely inactivating variants in tumor suppressor genes. Thus, with more than half of patient variants being classed as a VUS, clinical decisions or actions are often being made with fairly limited knowledge.

**Table 3:** Total number of distinct variants in each classification of interest is shown

Variant Classification	Distinct Variants	Total Counts
Novel	55,192	59,544
ExAC	6907	8237
Recurrent	4561	25,378
Intronic	1039	1205
Hotspot indels	1211	1459
Inactivating TSG	8834	10,294
GENIE recurrent	730	2598
VUS	56,032	60,195
Novel VUS	45,933	48,203
GENIE COSMIC recurrent	2586	5372

To better characterize these recurrent variants that are observed in many patient samples (Table 4), we leveraged additional information from COSMIC. Though before beginning special care was taken to remove potential artifacts originating from a single sequencing center pipeline by only considering variants reported by at least two sequencing centers. When first looking for recurrent variants appearing in at least three GENIE samples and not reported in COSMIC, we found 730 recurrent variants unique to GENIE. These variants appear in 1932 patient samples, or 10% of patients (Additional file 2: Table S2). The number of recurrent variants grows to 2586 affecting 3288 patients when pooling COSMIC and GENIE variant frequencies and still requiring they appear in at least three samples (Additional file 3: Table S3). While the proportion of cancer patients with these recurrent variants is relatively small at 10–20%, it still translates to millions of patients. For some, this information may lead to changes in the interpretation of their molecular profile and may affect diagnosis by altering disease subgrouping or lead to different treatment options. Though there is an expected decrease in the number of recurrent variants as the observation threshold increases, we still found that 4 variants appear more than 10 times in GENIE but fewer than three in COSMIC.

**Table 4:** Number of recurrent GENIE variants that are underrepresented in COSMIC (< 3 samples)

Minimum number of recurrences	GENIE recurrent variants	GENIE and COSMIC combined recurrent variants
10	4	5
9	4	7
8	5	15
7	15	44
6	40	100
5	87	264
4	236	740
3	730	2586

## Discussion

### COSMIC compatibility

With the intent of discovering new cancer-relevant variants from the GENIE data, we leveraged COSMIC as a point of reference for the current state of variant observation. A necessary consideration in such a comparison is the ability to map genes and variants between both resources. Across the 12 sequencing panels that comprise the GENIE dataset, 536 genes are mutated in GENIE samples. There was agreement between COSMIC and GENIE on most of the gene names and transcript ids with a few exceptions. For instance, PRKDC is the HGNC approved symbol [35]; however, COSMIC instead uses DNAPK. Additionally, CDK1’s canonical transcript is not defined in COSMIC. There were transcript compatibility issues for RUNX1T1, GNAS, DMD, and several other genes. For example, COSMIC picked ENST00000371085 (GNAS-015) with 394 amino acid residues as the canonical transcript whereas ENST00000371100 (GNAS-001) has 1037 amino acid residues. As a result, many variants can fall outside of COSMIC’s canonical transcript. Thus, we tried to rescue those variants by adapting the ENST00000371100 transcript as well. While in the GNAS case most variants could be rescued, 302 still fell outside of the COSMIC transcript. Recognizing the purpose of this study is to compare the GENIE variants with the standard COSMIC database, we opted not to rescue further variants.

### Unusual variants

Our analysis revealed a number of notable variants that had not previously been reported or were not observed at the same frequency in COSMIC. The frameshift variant EGFR L747 fs was found 13 times in GENIE but not once in COSMIC or ExAC. Although this particular variant was removed by the hotspot indels filter, we deemed it noteworthy because both its observed frequency is significantly higher than in COSMIC and it is an inactivating variant in a well-established oncogene. Indeed, as the variant occurs in the kinase domain, it would likely contribute to the truncation of that domain and the inactivation of the gene. Interestingly, it has been reported in literature that a patient harboring this variant has shown intermediate response to gefitinib (progression within 12 months) [36]. While at this point there remains the possibility that these are sequencing artifacts or the result of structural variants, such as amplification, the frequency with which they occur and the genes they fall within suggests their mechanisms warrant further study.

We also found several cases of variants likely leading to exon skip events. 13 variants were observed in the splice donor of MET exon 14 (c.3082 + 1 or c.3082 + 2). These variants are known to lead to MET exon 14 skipping events creating a constitutively active form of MET, and such patients were found to generally respond well to MET inhibitors, crizotinib and cabozantinib [37]. In addition to those splicing donor variants, we discovered an additional 17 variants in the coding region of the splicing donor. MET D1028H, MET D1028Y, and MET D1028N might also yield abnormal splicing similar to the exon 14 skipping variants. All D1028 variants were from NSCLC samples. These events should be confirmed with PCR or other methods before treatment with MET inhibitors.

### Highly recurrent variants

There are 40 novel, highly recurrent variants that are defined as appearing in more than 6 samples in the GENIE dataset and fewer than three in COSMIC (Table 5). The most frequent among them is MET A179T, which is found 19 times in GENIE and once in COSMIC. This variant has been reported in a chronic myelomonocytic leukemia patient but with no mention of its pathogenicity [38]. In GENIE the majority of samples in which it was detected were from NSCLC patients; although, all such samples were from a single sequencing center raising the possibility this particular variant is an artifact. Though as MET is already known to be frequently mutated in lung adenocarcinoma [39], study of this variant should likely be given priority.

**Table 5:** List of highly recurrent GENIE variants (≥ 6 samples) that are underrepresented in COSMIC (< 3 samples)

Gene	AA change	CDS change	GENIE count	COSMIC count	Cancer types
*MET*	p.A179T	c.535G > A	19	1	Non-Small Cell Lung Cancer(13);Melanoma(2);Colorectal Cancer(4)
ERBB3	p.E928G	c.2783A > G	14	2	Melanoma(1);Colorectal Cancer(2);Bladder Cancer(2);Hepatobiliary Cancer(1);Small Bowel Cancer(1);Breast Cancer(6);Esophagogastric Cancer(1)
CDKN2A	p.P75S	c.223C > T	13	1	Prostate Cancer(1);Colorectal Cancer(2);Ovarian Cancer(5);Non-Small Cell Lung Cancer(1);Endometrial Cancer(2);Breast Cancer(1);Cervical Cancer(1)
SMO	p.L23dup	c.67_69dupCTG	11	2	Thyroid Cancer(1);Melanoma(1);Glioma(2);Leukemia(2);Non-Small Cell Lung Cancer(2);Cancer of Unknown Primary(1);Endometrial Cancer(1);Breast Cancer(1)
SMARCA4	p.R1189Q	c.3566G > A	8	2	Glioma(1);Bladder Cancer(3);Renal Cell Carcinoma(1);Cancer of Unknown Primary(1);Breast Cancer(1);Esophagogastric Cancer(1)
CDKN2A	p.E69G	c.206A > G	7	0	Non-Small Cell Lung Cancer(6);Colorectal Cancer(1)
ERBB3	p.M91I	c.273G > A	7	2	Bladder Cancer(6);Endometrial Cancer(1)
ERBB3	p.K329E	c.985A > G	7	0	Bladder Cancer(4);Breast Cancer(1);Colorectal Cancer(1);Esophagogastric Cancer(1)
ERCC2	p.N238S	c.713A > G	7	0	Bladder Cancer(5);Breast Cancer(2)
FANCA	p.K1283R	c.3848A > G	7	0	Bladder Cancer(1);Endometrial Cancer(1);Hepatobiliary Cancer(1);Breast Cancer(3);Leukemia(1)
FLT1	p.R501K	c.1502G > A	7	0	Melanoma(1);Ovarian Cancer(1);Non-Small Cell Lung Cancer(2);Cancer of Unknown Primary(1);Breast Cancer(1);Cervical Cancer(1)
FLT4	p.P30fs	c.88delC	7	2	Non-Small Cell Lung Cancer(1);Colorectal Cancer(6)
IKZF1	p.D22N	c.64G > A	7	0	Colorectal Cancer(1);Non-Small Cell Lung Cancer(1);Melanoma(3);Glioma(1);Embryonal Tumor(1)
KDR	p.S265 L	c.794C > T	7	0	Myelodysplasia(1);Melanoma(1);Colorectal Cancer(1);Sellar Tumor(1);Non-Small Cell Lung Cancer(2);“Skin Cancer, Non-Melanoma”(1)
PIK3CB	p.R604fs	c.1809dupC	7	1	Bladder Cancer(1);“Skin Cancer, Non-Melanoma”(1);Breast Cancer(3);Endometrial Cancer(1);Esophagogastric Cancer(1)
APC	p.T1160K	c.3479C > A	6	0	Ovarian Cancer(1);Salivary Gland Cancer(1);Non-Small Cell Lung Cancer(1);Melanoma(1);Colorectal Cancer(2)
ARID1A	p.S735 N	c.2204G > A	6	0	Bladder Cancer(1);Hepatobiliary Cancer(1);Leukemia(1);Non-Small Cell Lung Cancer(1);Breast Cancer(1);Cervical Cancer(1)
ASXL1	p.S1028 L	c.3083C > T	6	1	Thyroid Cancer(1);Non-Small Cell Lung Cancer(2);Melanoma(1);Small Cell Lung Cancer(1);Prostate Cancer(1)
ATRX	p.G1071R	c.3211G > A	6	0	Skin Cancer, Non-Melanoma(1);Non-Small Cell Lung Cancer(2);Breast Cancer(1);Colorectal Cancer(1);Myeloproliferative Neoplasm(1)
BRCA1	p.E597K	c.1789G > A	6	1	Bladder Cancer(2);“Skin Cancer, Non-Melanoma”(1);Non-Small Cell Lung Cancer(1);Breast Cancer(1);Colorectal Cancer(1)
CARD11	p.R377Q	c.1130G > A	6	2	Non-Hodgkin Lymphoma(1);Colorectal Cancer(1);Uterine Sarcoma(1);Ovarian Cancer(1);Small Bowel Cancer(1);“Skin Cancer, Non-Melanoma”(1)
CDKN2A	p.V106 V	c.318G > A	6	2	Melanoma(1);Non-Small Cell Lung Cancer(2);Breast Cancer(1);Colorectal Cancer(1);Gastrointestinal Stromal Tumor(1)
ERBB4	p.E452K	c.1354G > A	6	2	Skin Cancer, Non-Melanoma(2);Melanoma(4)
ERCC3	p.R742W	c.2224C > T	6	1	Glioma(1);Sex Cord Stromal Tumor(1);Renal Cell Carcinoma(1);Non-Small Cell Lung Cancer(1);Leukemia(1);Colorectal Cancer(1)
FBXW7	p.R441W	c.1321C > T	6	1	Colorectal Cancer(3);Endometrial Cancer(1);Renal Cell Carcinoma(1);Esophagogastric Cancer(1)
IGF1R	p.R1246C	c.3736C > T	6	1	CNS Cancer(1);Endometrial Cancer(2);“Skin Cancer, Non-Melanoma”(1);Breast Cancer(1);Glioma(1)
IKZF1	p.E304K	c.910G > A	6	0	Melanoma(6)
KMT2D	p.K4832 N	c.14496G > T	6	0	Ovarian Cancer(1);Breast Cancer(1);Colorectal Cancer(2);Head and Neck Cancer(1);Thymic Tumor(1)
MET	p.D1028H	c.3082G > C	6	2	Non-Small Cell Lung Cancer(6)
MET	p.D1028Y	c.3082G > T	6	0	Non-Small Cell Lung Cancer(6)
MYC	p.S161 L	c.482C > T	6	0	Ovarian Cancer(1);Endometrial Cancer(2);Colorectal Cancer(2);Cervical Cancer(1)
PDCD1	p.T36 fs	c.104delC	6	2	Endometrial Cancer(2);Non-Small Cell Lung Cancer(1);Glioma(1);Colorectal Cancer(2)
PDCD1LG2	p.P81S	c.241C > T	6	0	Colorectal Cancer(1);Head and Neck Cancer(1);Appendiceal Cancer(1);Mesothelioma(1);Leukemia(1);Pancreatic Cancer(1)
PIK3C2B	p.G1435R	c.4303G > C	6	0	Head and Neck Cancer(1);Ovarian Cancer(1);Sellar Tumor(1);Appendiceal Cancer(1);Non-Small Cell Lung Cancer(1);Breast Cancer(1)
PMS2	p.K651R	c.1952A > G	6	1	Bladder Cancer(2);Uterine Sarcoma(1);Endometrial Cancer(1);Colorectal Cancer(2)
RAF1	p.S259F	c.776C > T	6	1	Small Bowel Cancer(1);Bladder Cancer(1);Non-Small Cell Lung Cancer(1);Melanoma(3)
ROS1	p.G1915R	c.5743G > A	6	0	Melanoma(1);“Skin Cancer, Non-Melanoma”(1);Non-Small Cell Lung Cancer(1);Breast Cancer(2);Cancer of Unknown Primary(1)
SMAD4	p.D351N	c.1051G > A	6	2	Appendiceal Cancer(1);Bladder Cancer(1);Hepatobiliary Cancer(1);Breast Cancer(1);Colorectal Cancer(2)
SMAD4	p.R361S	c.1081C > A	6	2	Pancreatic Cancer(1);Hepatobiliary Cancer(1);Colorectal Cancer(4)
SMAD4	p.G419 W	c.1255G > T	6	1	Colorectal Cancer(6)

The next most frequent variant is ERBB3 E928G. This particular variant has been experimentally confirmed to have higher activity and appears to activate EGFR allosterically upon heterodimerization [40, 41]. ERBB3 has two additional highly recurrent variants. The M91I variant appeared primarily in bladder cancer (6 of 7 samples), where it has been previously reported though its pathogenicity remains unknown. K329E variant was observed in seven samples, and four were endometrial cancer. Another ERBB family member variant, ERBB4 E452K, appeared mainly in skin cancers and has been confirmed to increase activity [42].

The cell cycle regulating protein, CDKN2A, is frequently inactivated in various cancer types. While COSMIC there are several variants occurring at CDKN2A P75 residue, such as P75L and P75S, that are reported only once, we observed them 13 times in GENIE. CDKN2A P75L has been functionally studied and concluded to be benign [43]. Another CDKN2A variant, E69G, takes places mostly in NSCLC. Although E69G is never observed in COSMIC, other codon E69 variants have been reported there. E69G was observed in GENIE as belonging mostly to NSCLC samples. There have been reports of CDKN2A E69G in familial melanoma patients with 30% decreased binding to CDK4 compared with its wild type [44]. The CDKN2A variant, V106 V, is a synonymous mutation for CDKN2A; however, the same locus is used for protein p14 (ARF), which is a tumor suppressor. This mutation translates to p14(ARF) A162T.

SMO L23dup (or L23_G24insL in COSMIC notation) was found 11 times in GENIE but only twice in COSMIC. This variant, along with two other detected variants (L23_G23insLL and L23_G23insA), resides in a signal peptide domain found in the first 27 residues. SMO L23dup was previously reported in a mesothelioma cell line LO68 and two gastric cancer patients; however, no functional significance was observed but it might affect processing of SMO precursor [45]. Though this alteration was detected in GENIE in a diverse array of cancers, there is potential for it to be a sequencing artifact because it originated from only a single sequencing center.

Variants in the SWI/SNF components, ARID1A S735 N and SMARCA4 R1189Q, were also found to be highly recurrent. SMARCA4 R1189Q has been reported in 2 COSMIC samples, and in GENIE, 3 of 7 samples were bladder cancer. There are not yet reports on pathogenicity regarding these two variants. It may be possible to assess whether these variants in SWI/SNF genes contribute to tumorigenesis by studying epigenetic signatures using techniques like ATAC-seq [46].

FBXW7 is a ubiquitin ligase and known to function as a tumor suppressor regulating NOTCH, MYC, and other oncogenes [47, 48]. FBXW7 is frequently mutated in colorectal cancer. FBXW7 R441W appears 3 times out of 6 in colorectal cancer and is located near R465, R479, and R505 hotspots. There are currently no reports in literature for this particular variant. Although the FBXW7 variant is not generally considered actionable, FBXW7 is one of the most mutated genes in cancer and developing sensitivity or resistance information related the variant would be beneficial.

DNA repair genes BRCA1, ERCC2, ERCC3, and FANCA are known to affect responses to chemotherapeutic agents and PARP1 inhibitors. ERCC2 N238S was observed seven times in GENIE and five of those samples were bladder cancer. ERCC2 variants are also known to improve response to platinum agents [49]. These ERCC2 variants could prove informative for changing the outcome of certain patients by serving as a therapeutic biomarker. FANCA K1283R appeared three times in breast cancer out of seven cases. FANCA variants have been reported in non-BRCA1/2 familial breast cancer patients [50]. FANCA’s role in homologous recombination suggests that patients with loss of function variants might be susceptible to PARP1 inhibitor treatment [51]. While BRCA1 is obviously an important cancer gene, the clinical significance of recurrent BRCA1 E597K variant is not yet known.

FLT4 frameshift variant P30fs was observed in colorectal cancers in 6 of the 7 samples it appeared, and the 2 COSMIC reported cases were also colorectal cancer. Given FLT4’s believed function as an oncogene playing a role in invasion and metastasis [52], further investigation should be made as to the relevancy of this variant or FLT4’s role in pathogenicity in colorectal cancer. This might also indicate a potential tumor suppressor role for FLT4 gene in colorectal cancer. Another FLT family member variant FLT1 R501K was found to be highly recurrent. FLT1 is a VEGF receptor along with KDR (VEGFR2), which also had a highly recurrent variant in GENIE, S265 L. Neither FLT1 R501K nor KDR S265 L have confirmed pathogenicity.

SMAD4 has three recurrent variants D351N, R361S, and G419 W. SMAD4 is one of the most mutated genes in colorectal cancer. Considering its high occurrence in colorectal cancer, these variants may reduce activity of SMAD4 and contribute to the development of colorectal cancer. Along with SMAD4, APC is another important gene in colorectal cancer. APC T1160K appeared in colorectal cancer for 2 out of 6 samples it was found. At this point, none of these variants have confirmed pathogenicity.

Many variants in IKZF1 are observed in melanoma. Three of the 7 samples D22N was found and all 6 samples where E304K was detected originated from melanoma samples. The relationship between IKZF1 and melanoma is not yet well established. However, it was recently reported that IKZF1 expressing cells respond better to PD-1/CTLA-4 [53]. These variants in IKZF1 along with PDCD1 (PD-L1) T36 fs and PDCD1LG2 (PD-L2) P81S should be investigated for response to PD-1/CTLA-4 inhibitors.

There are various kinases – IFI1R, PIK3C2B, ROS1, and RAF1 – in the set of highly recurrent variants. Although BRAF has gained more attention in melanoma, RAF1 plays an important role in MAPK signaling. The RAF1 S259 residue is critical to bind the inhibitory 14–3-3 protein [54]. Since 3 of the 6 samples that possessed RAF1 S259F were melanoma, this variant may contribute to melanoma development.

## Conclusions

While our variant analysis of the GENIE dataset focusing on VUS’s is only beginning to scratch the surface, it does provide a more comprehensive assessment of the landscape of cancer variants. Many of these VUS’s require additional study to disentangle their roles in cancer formation and progression. Yet, using the frequencies with which they occur and how they are distributed among cancer types, this analysis can already aid clinicians working to develop a course of treatment. Currently, there are significant disparities in the reporting of variants. For instance, there are thousands of papers concerning BRAF V600E and EGFR L858R, but many of the most frequent variants registered in COSMIC are not published in a journal article. COSMIC contains 2 million unique coding variants, and it is not practical to publish articles on all equally. However, the recurrent variants revealed in this study are good candidates for further research. There exist several reasons, both technical and biological, for the differences between our findings in GENIE and that of COSMIC. The technical reasons include differences in platforms, reagents, and data processing pipelines. The biological differences may be partly attributable to ethnic and regional sampling differences. For instance, chemical and microbial exposure can vary greatly region to region. Well-coordinated strategies to cover these variants must be developed to mitigate such differences, to efficiently deploy scientific resources, and to overcome the lack of coverage in the published literature. Only with these persistent efforts will the clinical utility of precision medicine be fully demonstrated.

## Additional files

Additional file 1: **Table S1.** List of Genes in GENIE. -The file contains genes in GENIE with transcripts used both in GENIE and COSMIC. Each tumor suppressor gene is marked with evidences in Pubmed ID. This information was used to identify inactivating variants in tumor suppressor genes. (XLSX 34 kb)

Additional file 2: **Table S2.** Variants recurring more than 3 times in GENIE samples only. Variants are ordered by the recurrence counts in GENIE. For each variant, AA change and CDS change are shown with COSMIC and GENIE sample counts. Z-value was computed for each variant using ExAC information (ExAC_AC, ExAC_AN, sample number). The table also contains sample IDs and cancer types for each variant. (XLSX 83 kb)

Additional file 3: **Table S3.** Variants recurring more than 3 times in GENIE and COSMIC samples combined. Variants are ordered by the combined recurrence counts in GENIE and COSMIC. . For each variant, AA change and CDS change are shown with COSMIC and GENIE sample counts. Z-value was computed for each variant using ExAC information (ExAC_AC, ExAC_AN, sample number). The table also contains sample IDs and cancer types from both GENIE and COSMIC. (XLSX 287 kb)

## Abbreviations

AACR: American Association for Cancer Research
CDS: coding sequence
COSMIC: Catalogue Of Somatic Mutations In Cancer
ExAC: Exome Aggregation Consortium
FDA: Food and Drug Administration
GENIE: Genomics Evidence Neoplasia Information Exchange
NSCLC: non-small cell lung cancer
VUS: variant of unknown significance
